# Phylogenetic data reveal a surprising origin of *Euphorbia orphanidis* (Euphorbiaceae) and environmental modeling suggests that microtopology limits its distribution to small patches in Mt. Parnassus (Greece)

**DOI:** 10.3389/fpls.2023.1116496

**Published:** 2023-02-16

**Authors:** Felix Faltner, Johannes Wessely, Božo Frajman

**Affiliations:** ^1^ Department of Botany, University of Innsbruck/Universität Innsbruck, Sternwartestrasse, Innsbruck, Austria; ^2^ Department of Botany and Biodiversity Research, University of Vienna, Rennweg, Vienna, Austria

**Keywords:** endemism, endangered species, environmental modelling, Mediterranean basin, morphometry, phylogeny, taxonomy

## Abstract

The Mediterranean Basin is one of the most biodiverse areas in the world, harboring 25,000 plant species, of which 60% are endemic. Some of them have narrow distributions, such as *Euphorbia orphanidis*, which is only known from alpine screes on Mt. Parnassos in Greece. Its exact distribution in this mountain was, however, poorly known, and its phylogenetic origin was also unclear. We performed extensive field work in Mt. Parnassos and could register *E. orphanidis* only in five patches of limestone screes in the eastern part of this mountain range, emphasizing its very narrow distribution, which is likely limited by topography influencing water availability as indicated by environmental modeling. We also registered 31 accompanying species and thus characterized its habitat. Using nuclear ribosomal internal transcribed spacer and plastid *ndhF–trnL* and *trnT–trnF* sequences, we show that it belongs to *E.* sect. *Patellares*, despite not having connate raylet leaves typical for this section, and not to *E.* sect. *Pithyusa* as previously suggested. The relationships among the species of *E.* sect. *Patellares* are poorly resolved, suggesting their simultaneous divergence that dated to the late Pliocene, which coincided with the establishment of the Mediterranean climate. The relative genome size of *E. orphanidis* is in the range of that for the other members of *E.* sect. *Patellares*, suggesting that it is diploid. Finally, we performed multivariate morphological analyses to generate a comprehensive description of *E. orphanidis*. Based on its narrow distribution and the anticipated negative impact of global warming, we consider this species endangered. Our study demonstrates how microrelief can limit the distribution of plants in topographically heterogeneous mountain environments and likely plays an important, yet neglected, role in shaping the distribution patterns of plants in the Mediterranean Basin.

## 1 Introduction

The Mediterranean Basin is one of the richest areas in the world in terms of animal and plant diversity and is considered one of the 25 global biodiversity hotspots (Myers et al., 2000). Owing to its geological and climatic complexity conferring a unique mosaic of habitats, it hosts approximately 25,000 plant species, of which 60% are endemic (Cuttelod et al., 2008; Nieto Feliner, 2014). One of the most biodiverse countries in the Mediterranean is Greece, which is positioned at the crossroads between Europe and Asia, harboring roughly 5,800 species of vascular plants. Of these, 1,462 (22%) are endemic (Dimopoulos et al., 2013), with the majority being restricted to small areas (Georghiou and Delipetrou, 2010). Their highest richness is in the Peloponnesus (468 taxa), followed by Crete and Karpathos (395 taxa), and Sterea Ellas, positioned just north of the Golf of Corinth, with 368 taxa (Dimopoulos et al., 2013). Local endemics in the coastal areas and the islands are predominantly distributed at low elevations (0–600 m a.s.l.), whereas those in the continental Greece occur mostly at higher elevations (Georghiou and Delipetrou, 2010). Many of them are linked to rock crevices and screes (Tan et al., 2001), which are particularly sensible to human disturbances (Panitsa et al., 2021).

Different factors, such as topographic, geologic, and climatic conditions, influence the distribution of endemic species (Fois et al., 2017; Zangiabadi et al., 2021). Narrow endemics are generally less stress tolerant than their widespread relatives and have specific ecological requirements. They often grow on steep slopes with high rock cover, such as screes, and in more open vegetation than their widespread congeners (Lavergne et al., 2004). Topographical variability that is particularly complex in mountainous regions can influence climatic and other plant growing conditions that strongly influence species distribution at a local scale (Whittaker and Levin, 1977; Ackerly et al., 2010; Boehm et al., 2021; Man et al., 2022). Such distinct habitats are often associated with restricted ranges of environmental conditions that affect species survival, but the exact causes limiting the distribution of narrow endemics are still poorly understood (Manne and Pimm, 2001; Munson and Sher, 2015).

One of the narrow endemics from high elevations in Sterea Ellas in Greece is *Euphorbia orphanidis* Boiss (Dimopoulos et al., 2013). It was first collected by Theodorus Orphanides near Lugari on Mt. Parnassos in 1852 and distributed in 1854 as *E. hohenackeri* within Flora Graeca Exiccata (no. 407). As the name *E. hohenackeri* Hochst. et Steud. was earlier applied for another species, it was renamed *E. orphanidis* by Boissier (1859). Since then, it has only been collected (see *Specimina visa*) and reported (Quezel, 1964; Garnweidner, 1986) a few times from calcareous rocks and screes on Mt. Parnassos, but despite its narrow range, its exact distribution remains unknown (Parnassos National Park Authority, personal communication). It is considered to grow from the upper part of the montane coniferous forests dominated by the Greek endemic *Abies cephalonica* Loudon and *Pinus nigra* J.F. Arnold (1,500 m) to the alpine zone (2,100 m; Aldén, 1986). Along with *E. orphanidis*, many other rare and endemic species, such as *Astragalus parnassia* Boiss., *Colchicum parnassicum* Sart.Orph. & Heldr., *Convolvulus parnassicus* Boiss. & Orph., *Paeonia parnassica* Tzanoud., and *Scutellaria rupestris* subsp. *parnassica* (Boiss.) Greuter & Burdet, occur in Mt. Parnassos. This mountain has an outstanding position among the Greek mountains and is regarded as a hotspot of endemic taxa, with several of them being critically endangered (Kougioumoutzis et al., 2021). The high concentration of rare endemics is a result of topological heterogeneity and the geographical position of Mt. Parnassos between the biodiversity hotspots of Peloponnesus and Pindos Mountains (Kougioumoutzis et al., 2021) and led to the foundation of the National Park Parnassos in 1938 (Tsitsoni et al., 2015). However, despite its limited distribution, *E. orphanidis* itself is not a protected species in Greece.

*Euphorbia orphanidis* is a glabrous, glaucous, prostrate to ascending perennial with an extensive, branched, fleshy rhizome and stems rising up to 15 cm (Radcliffe-Smith and Tutin, 1968). Based on its morphology, it was included in *Euphorbia* sect. *Paralias* Dumort. subsect. *Conicocarpae* (Prokh.) Prokh. by Geltman (2009) and correspondingly in *Euphorbia* sect. *Pithyusa* (Raf.) Lázaro in the most recent taxonomic treatment by Riina et al. (2013), but its phylogenetic position remains unknown. The latter section includes 50, mainly glabrous and glaucous species, often growing on rocky calcareous grounds, with a high diversity in the Mediterranean (Riina et al., 2013).

Considering the unknown phylogenetic position as well as the poorly known distribution of *E. orphanidis*, our aims were to investigate the evolutionary origin and to determine the systematic position of this species. In addition, we gathered exact data about its distribution and ecology and thus provide the information needed to design future conservation strategies as well as to disentangle environmental factors that possibly limit its distribution. To achieve the first aim, we (1) sequenced nuclear ribosomal internal transcribed spacer (ITS) and plastid *trnT*–*trnF* (*trnTF* in the following) and *ndhF–trnL* to infer its phylogenetic position with a dense sampling of closely related species, (2) estimated its relative genome size (RGS) using flow cytometry and compared it to the RGS of closely related species, and (3) determined its morphological characteristics using multivariate morphometrics. To achieve the second aim, we (4) performed extensive field work on Mt. Parnassos in 2019 and 2020 and mapped the occurrences of *E. orphanidis*, (5) determined its accompanying species, and (6) performed environmental niche modeling by applying different topographic predictors that likely determine the distribution range of *E. orphanidis* on Mt. Parnassos. We finally (7) assessed the conservation status of this narrow endemic following the IUCN criteria.

## 2 Materials and methods

### 2.1 Plant material

**Figure 1:**
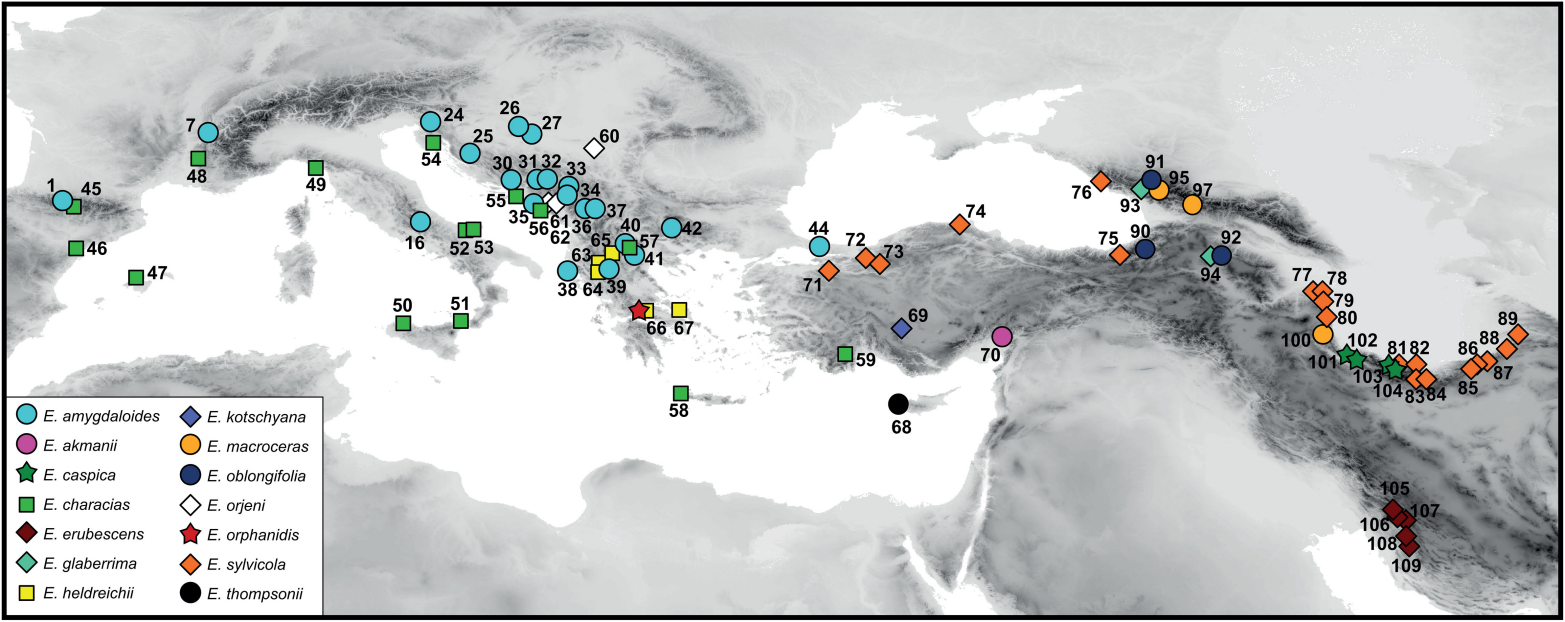
Distribution of populations of *Euphorbia* sect. *Patellares* used in phylogenetic and partly relative genome size analyses. The population numbers correspond to **Supplementary Table S1** and are not indicated for *Euphorbia orphanidis*.

The plant material of *E. orphanidis* for RGS estimation and molecular and morphometric analyses was sampled from seven localities on Mt. Parnassos in 2020. For molecular and RGS analyses, leaf material was collected in silica gel, and herbarium vouchers were pressed. The morphological characteristics of *E. orphanidis* were studied on the 18 specimens that we collected (**Supplementary Table S1**) as well as on seven specimens from six collections deposited in the herbaria G, M, W, and WU, indicated by asterisks following the herbarium IDs in the “Taxonomic treatment” section. In addition, 13 outgroup species from the same section (109 populations) as well as 27 species from 10 closely related sections were included in the phylogenetic analyses; species from the same section were also subjected to RGS analyses (**Supplementary Tables S1**, **S2**). The ITS and *ndhF–trnL* sequences of most of accessions besides *E. orphanidis* were taken from previous studies (mostly from Pahlevani and Frajman, 2023), whereas most of the *trnTF* sequences were generated for this study (**Supplementary Tables S1**, **S2**; **Figure 1**).

### 2.2 Field work: Mapping the distribution of *E. orphanidis* and recording accompanying species

We visited Mt. Parnassos in September 2019 and 2020 and mapped the presence of *E. orphanidis* across the mountain range. We visited the localities given in the literature (Quezel, 1964; Garnweidner, 1986) as well as those from the herbarium labels. After acquiring knowledge about the ecology of this species, we systematically screened the majority of the screes in the eastern part of the mountain range as well as some in the central part indicated by Garnweidner (1986) and mapped the presence (or absence) of the species. In addition, we registered the accompanying species of *E. orphanidis* that were identified using Strid (1986), Strid and Tan, (1986), and Papiomytoglou et al. (2021).

### 2.3 DNA extraction, sequencing, and analyses of sequence data

Extraction of total genomic DNA and sequencing were performed for ITS and *trnTF* as described by Frajman and Schönswetter (2011) and for *ndhF–trnL* by Pahlevani and Frajman (2023). The sequencing was carried out at Eurofins Genomics (Ebersberg, Germany). Contigs were assembled and edited, and sequences were aligned using Geneious Pro 5.5.9 (Kearse et al., 2012). As the preliminary analyses showed that *E. orphanidis* belongs to *E.* sect. *Patellares* (Prokh.) Frajman and not to *E.* sect. *Pithyusa*, we sampled several accessions of different species from the former section. In total, 115 ITS, 73 *ndhF–trnL*, and 41 *trnTF* sequences were used, partly from previous studies and partly newly generated (**Supplementary Tables S1**, **2**). Maximum parsimony (MP) and MP bootstrap (MPB) analyses were performed using PAUP 4.0b10 (Swofford, 2002). The most parsimonious trees were searched for heuristically with 100 replicates of random sequence addition, TBR swapping, and MulTrees on. The swapping was performed on a maximum of 1,000 trees (nchuck = 1,000). All characters were equally weighted and unordered. The data set was bootstrapped using full heuristics, 1,000 replicates, TBR branch swapping, MulTrees option off, and random addition sequence with five replicates. The Bayesian analyses were performed using MrBayes 3.2.1 (Ronquist et al., 2012), applying the GTR+Γ substitution model proposed by the Akaike information criterion implemented in MrAIC.pl 1.4 (Nylander, 2004) for all datasets. Values for all parameters, such as the shape of the gamma distribution, were estimated during the analyses. The settings for the Metropolis-coupled Markov chain Monte Carlo process included four runs with four chains each (three heated ones using the default heating scheme) and run simultaneously for 10,000,000 generations each, with sampling trees every 1,000th generation using default priors. The posterior probabilities (PP) of the phylogeny and its branches were determined from the combined set of trees, discarding the first 1,001 trees of each run as burn-in. Tracer 1.6 (Rambaut et al., 2014). was used to assess convergence and mixing, which were appropriate. In addition, a NeighborNet was produced with ITS sequences of *E.* sect. *Patellares*, using SplitsTree 4.12.3 (Huson and Bryant, 2006).

Divergence times were estimated with BEAST 1.8.2 (Drummond et al., 2012) using a pruned ITS alignment containing 22 accessions of *E.* sect. *Patellares* and 15 accessions of its sister clade inferred with the analysis of the entire ITS dataset. Birth–death speciation prior (Gernhard, 2008) and GTRI+Γ substitution model with estimated base frequencies were used for the phylogeny inference. A lognormal relaxed clock with a weakly informative prior on the clock rate (exponential with mean 0.001) was applied. The prior age of the root was set to 17.7 million years with a normally distributed standard deviation of 2.3, which corresponds to the median age and 95% highest posterior densities (HPD) interval of the corresponding node (17.7 My; HPDs, 13.2–22.6; *i*.*e*., the split between *E.* sect. *Patellares* and its sister clade) obtained from the dating analysis by Horn et al. (2014). Two independent MCMC chains were run for 10,000,000 generations, saving trees and parameters every 1,000 generations. The performance of the analysis was checked with Tracer 1.6 (Rambaut et al., 2014); both the effective sample sizes (ESS >200) and mixing were appropriate. The log and tree files from both runs were combined using Log Combiner (part of the BEAST package) after discarding 10% of each run as burn-in. The maximum clade credibility tree was then produced and annotated with Tree Annotator (part of the BEAST package) and visualized with FigTree 1.4.2 (Rambaut, 2014).

### 2.4 Relative genome size measurements

The RGS of 25 individuals of *E. orphanidis* from five localities were measured with a CyFlow space flow cytometer (Partec, GmbH, Münster, Germany) using 4′,6-diamidino-2-phenylindole and the reference standard *Bellis perennis* L. (2C = 3.38 pg; Schönswetter et al., 2007) following Suda and Trávníček (2006) and the modifications described by Cresti et al. (2019). In addition, the RGS values of 68 populations belonging to eight species from the same section as *E. orphanidis* were included in the analyses for comparison. The RGS data were visualized in RStudio 1.2.5001 (RStudio Team, 2019) by utilizing R-3.4.0 and the package “ggplot2”.

### 2.5 Morphometric analyses

A total of 24 individuals from different collections (those collected by us are shown in **Supplementary Table S1** and those from other collections are marked with an asterisk in the “Taxonomic treatment” section) were analyzed morphometrically. A total of 36 metric characters were measured or scored; based on them, 17 ratio characters were calculated (see the species description below). Plant height, stem, leaf, and ray characters were measured/scored manually. All the other characters (cyathium, fruit, and seed characters) were measured on microscopic images taken with a stereomicroscope Olympus SZX9 using the Olympus image analysis software analySIS pro.

### 2.6 Environmental modeling

**Figure 2:**
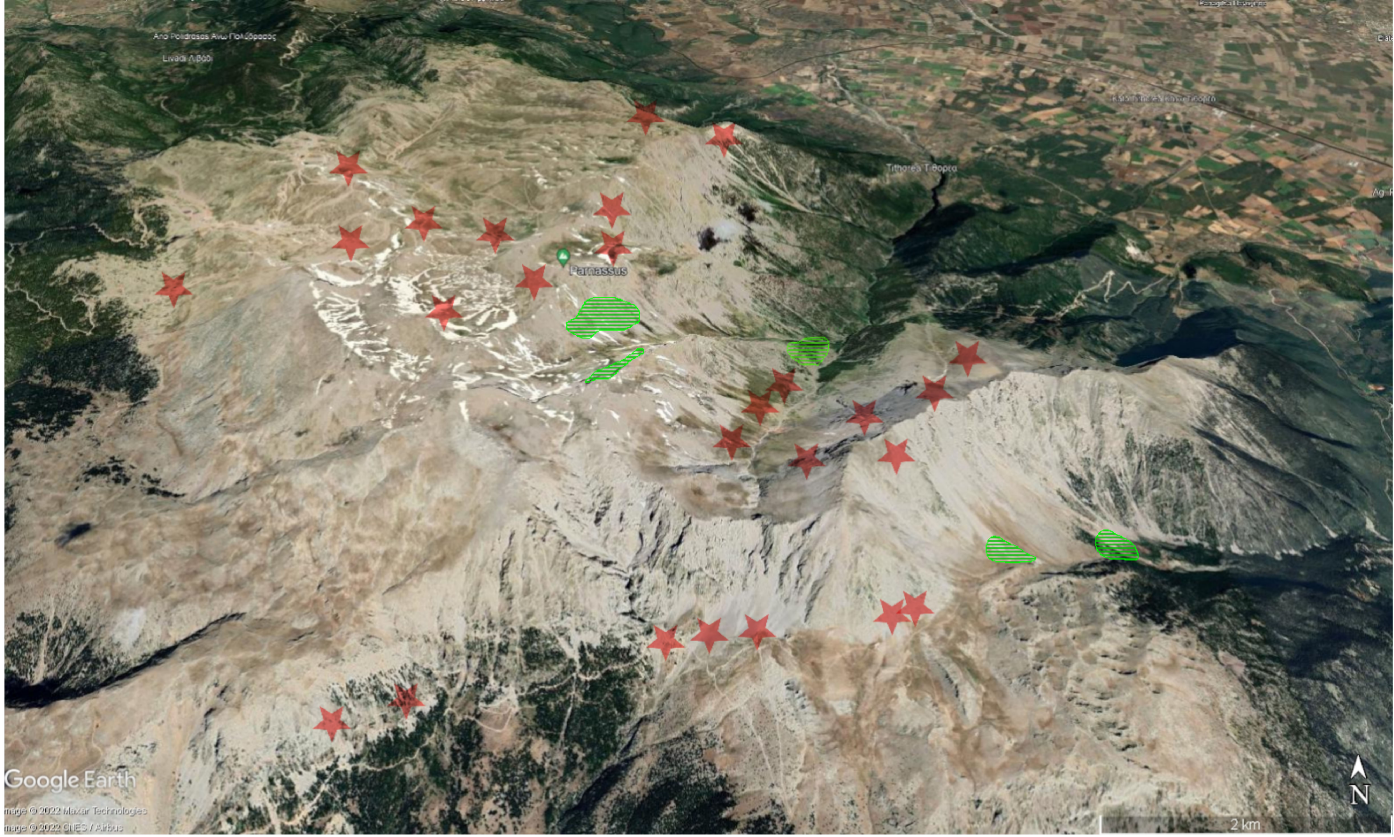
Distribution of *Euphorbia orphanidis* on Mt. Parnassus (green), with indicated areas as to where the species was searched for but not found (red stars). The map was adopted from Google Earth .

As the presence/absence data of *E. orphanidis* were unbalanced (seven presences and 37 absences), we used a resampling approach to evaluate the potential influence of topographic predictors. We therefore resampled as many absences as presences from the dataset 10,000 times. For these samples, we derived the mean of all predictors and evaluated if the mean of the presences was outside the 95% confidence interval (see **Figure 2**). As topographic predictors, we used the Terrain Ruggedness Index (TRI), Topographic Position Index (TPI; Wilson et al., 2007), roughness, slope, aspect, and flow direction (flowdir), *i*.*e*., the direction of the greatest drop in elevation. TRI is a measure of ruggedness, expressing the amount of elevation difference between adjacent cells. TPI, on the other hand, is a measure for terrain classification. It compares the elevation of a cell to the mean elevation of the surrounding cells. Hence, a negative value represents a cell that is lower than its surroundings (valleys). We did not evaluate climatic predictors due to the small extent covered by the occurrence data; for such small regions, the available climate data consists of measurements of the nearest climate station, which is statistically downscaled *via* correlations to altitude. Therefore, climatic variation in the data would only reflect elevational variation.

## 3 Results

### 3.1 ITS and plastid *ndhF–trnL* and *trnTF* phylogenies

**Figure 3:**
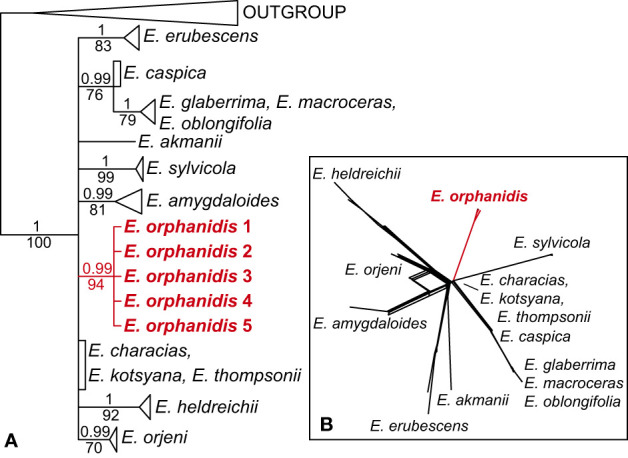
Phylogenetic relationships inferred from internal transcribed spacer sequences among the members of *Euphorbia* sect. *Patellares*. **(A)** Bayesian consensus phylogram; the numbers above the branches are posterior probability values >0.5, while those below the branches are maximum parsimony bootstrap values >50%. The clades of all species including multiple accessions, except that of *Euphorbia orphanidis*, are collapsed, and the complete tree is shown in **Supplementary Figure S1**. **(B)** NeighborNet.

The ITS sequences of *E. orphanidis* were 693, and the alignment was 739 characters long. A total of 191 characters (25.8%) were parsimony-informative. The homoplasy index was 0.37 (0.43 after exclusion of uninformative characters), and the retention index was 0.86. A total of 100,000 most parsimonious trees were found, and their score was 545. The Bayesian and maximum parsimony reconstructions resulted in congruent topologies when considering clades with MPB >70% and PP >0.95 (**Figure 3A**; **Supplementary Figure S1**). *Euphorbia* sect. *Patellares* was monophyletic (PP 1, MPB 100%), and within the section single accessions of *E. akmanii* I. Genç & Kültür*, E. kotschyana* Fenzl and *E. thompsonii* Holmboe and multiple accessions of *E. characias* L. were included in a basal polytomy, in which several monophyletic clades, mostly corresponding to species, were also included. One of them corresponded to *E. orphanidis* (PP 1, MPB 99%) and the others to *E. heldreichii* Orph. ex Boiss. (PP 1, MPB 92%) and *E. orjeni* Beck (PP 0.99, MPB 70%), which together formed a poorly supported clade in the parsimony tree (MPB 67%) as well as *E. amygdaloides* L. (PP 0.99, MPB 81%), *E. erubescens* Boiss. (PP 1, MPB 83%), *E. sylvicola* Pahlevani & Frajman (PP 1, MPB 99%), and a clade (PP 0.99, MPB 76%) including *E. caspica* Frajman & Pahlevani in a basal polytomy and a clade (PP 1, MPB 79%) of *E. glaberima* K. Koch, *E. macroceras* Fisch. & C.A. Mey, and *E. oblongifolia* (K. Koch) K. Koch. The clade including *E. caspica*, *E. glaberima, E. macroceras*, and *E. oblongifolia* was resolved as sister to a clade (MPB 57%) including all other species of *E.* sect. *Patellares* by parsimony, but not Bayesian analysis. Consistent with the ITS tree, the NeighbourNet of *E.* sect. *Patellares* (**Figure 3B**) was star-like, with *E. characias, E. kotsyana*, and *E. thompsonii* in the center of the star and *E. amygdaloides*, *E. akmanii*, *E. erubescens*, *E. heldreichii*, *E. orjeni*, *E. orphanidis*, and *E. sylvicola* in the terminal parts of their own star-rays. *Euphorbia glaberrima*, *E. macroceras*, and *E. oblongifolia* were all in the terminal part of a ray, in which *E. caspica* was positioned in the central part.

**Figure 4:**
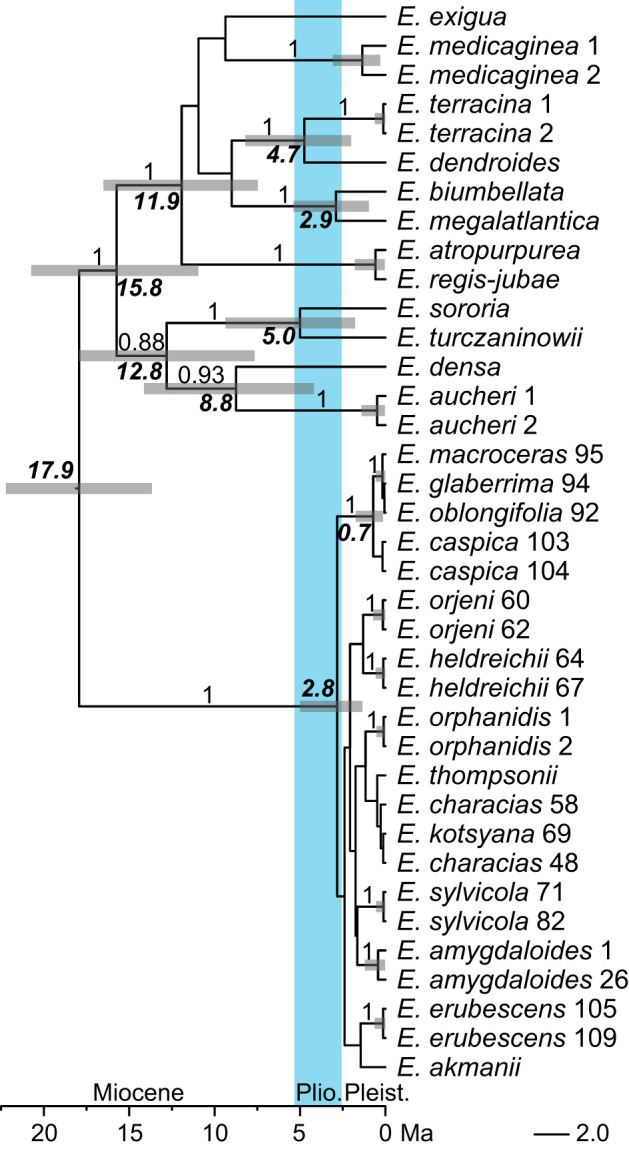
Bayesian consensus chronogram (Maximum Clade Credibility tree) inferred from internal transcribed spacer sequences. The numbers above the branches are posterior probability values >0.90, the bold numbers associated with the nodes indicate the median crown group ages in millions of years of the clades diversifying at those nodes, and the bars correspond to 95% highest posterior density of the age estimates. The population numbers of *Euphorbia* sect. *Patellares* correspond to the data in **Supplementary Table S1** and **Figure 1**, while those of the outgroup sections correspond to the data in **Supplementary Table S2**.

The topology of the ITS chronogram (**Figure 4**) was congruent with that of the ITS tree (**Figure 3A**), *i*.*e*., there was a polytomy of several clades mostly corresponding to species since all other clades resolved in the tree had low support (PP < 0.8). *Euphorbia* sect. *Patellares* originated in the Miocene, 17.9 Ma (HPDs: 13.7–22.2 Ma), whereas the onset of its diversification was dated to the late Pliocene, 2.8 (1.3–5.5) Ma. This was also the time of the origin of all main lineages (mostly corresponding to species) within *E.* sect. *Patellares*, whereas their diversification was dated to the Pleistocene.

**Figure 5:**
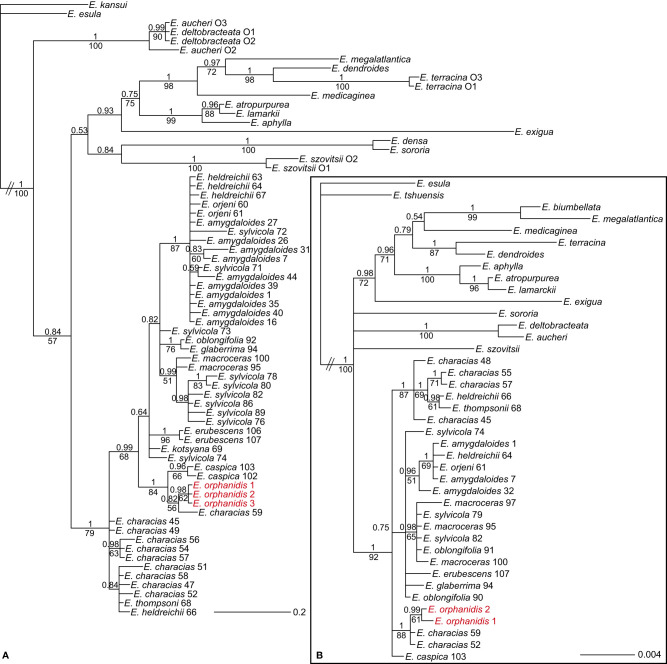
Bayesian consensus phylograms of plastid *ndhF–trnL* **(A)** and *trnTF* **(B)** sequences showing the phylogenetic relationships among the members of *Euphorbia* sect. *Patellares* and outgroup species. The numbers above the branches are posterior probability values >0.5, while those below the branches are maximum parsimony bootstrap values >50%. The population numbers of *E.* sect. *Patellares* correspond to the data in **Supplementary Table S1** and **Figure 1**, while those of the outgroup sections correspond to the data in **Supplementary Table S2**.

The *ndhF–trnL* sequences of *E. orphanidis* were 911, and the alignment was 1,229 characters long. A total of 130 characters (10.6%) were parsimony-informative. The homoplasy index was 0.19 (0.29 after exclusion of uninformative characters), and the retention index was 0.89. In total, 100,000 most parsimonious trees were found, and their score was 349. The Bayesian and maximum parsimony reconstructions resulted in congruent topologies (**Figure 5A**). *Euphorbia* sect. *Patellares* was monophyletic (PP 1, MPB 79%), the relationships within the section were poorly resolved, and several species appeared polyphyletic. In the basal polytomy, several accessions of *E. characias* (three grouped together in a clade) and one accession each of *E. heldreichii* and *E. thompsonii* were included, along with a clade (PP 0.99, MPB 68%) including all other accessions. In this clade, relationships were also poorly resolved, with several accessions in a basal polytomy (considering the clades with support values PP 0.64 and 0.82 as non-relevant) or in small clades including only two accessions, like that of *E. erubescens* (PP 1, MPB 96%). There were three bigger clades in this polytomy. The first (PP 1, MPB 84%) included *E. caspica* (PP 0.96, MPB 66%) and a poorly supported clade (PP 0.82, MPB 56%) with one accession of *E. characias* from westernmost Anatolia and a clade (PP 0.98, MPB 62%) including *E. orphanidis*. The second (PP 0.99, MPB 51%) included *E. macroceras* and *E. sylvicola*, and the third (PP 1, MPB 87%) consisted of *E. amygdaloides*, *E. heldreichii*, *E. orjeni*, and *E. sylvicola*.

The *trnTF* sequences of *E. orphanidis* were 1,486, and the alignment was 1,869 characters long. A total of 97 characters (5.2%) were parsimony-informative. The homoplasy index was 0.09 (0.18 after exclusion of uninformative characters), and the retention index was 0.91. Moreover, 97,000 most parsimonious trees were found, and their score was 262. The Bayesian and maximum parsimony reconstructions resulted in congruent topologies (**Figure 5B**) that mostly corresponded to the topology of the *ndhF–trnL* tree, although some differences could be observed. Contrary to the *ndhF–trnL* tree, *E. caspica* was not in the same clade as *E. orphanidis*, and in addition to population 59 of *E. characias* from Turkey, population 52 of *E. characias* from Italy was also in this clade (PP 1, MPB 88%); the latter population was in the basal polytomy of *E.* sect. *Patellares* along with some other accessions of *E. characias* and one each of *E. heldreichii* and *E. thompsonii* in the *ndhF–trnL* tree, if considering PP 0.84 non-relevant.

### 3.2 Relative genome size

**Figure 6:**
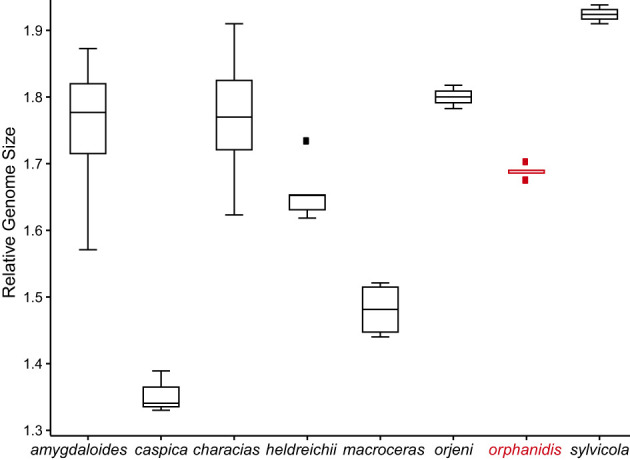
Relative genome size (RGS) variation in *Euphorbia orphanidis* and other species of *Euphorbia* sect. *Patellares*. The boxplots are based on the RGS population mean values presented in **Supplementary Table S1**. A scatterplot representing the mean values and standard deviation of all investigated populations is shown in **Supplementary Figure S2**.

The relative genome size of *Euphorbia orphanidis* varied between 1.675 and 1.703, which was in the range of the RGS of *E. amygdaloides* (1.571–1.873), *E. characias* (1.623–1.910), and *E. heldreichii* (1.619–1.734), whereas *E. caspica*, *E. macroceras*, *E. orjeni*, and *E. sylvicola* had a deviating RGS (**Figure 6**; **Supplementary Figure S2**; **Supplementary Table S1**).

### 3.3 Distribution of *E. orphanidis* on Mt. Parnassos

**Figure 7:**
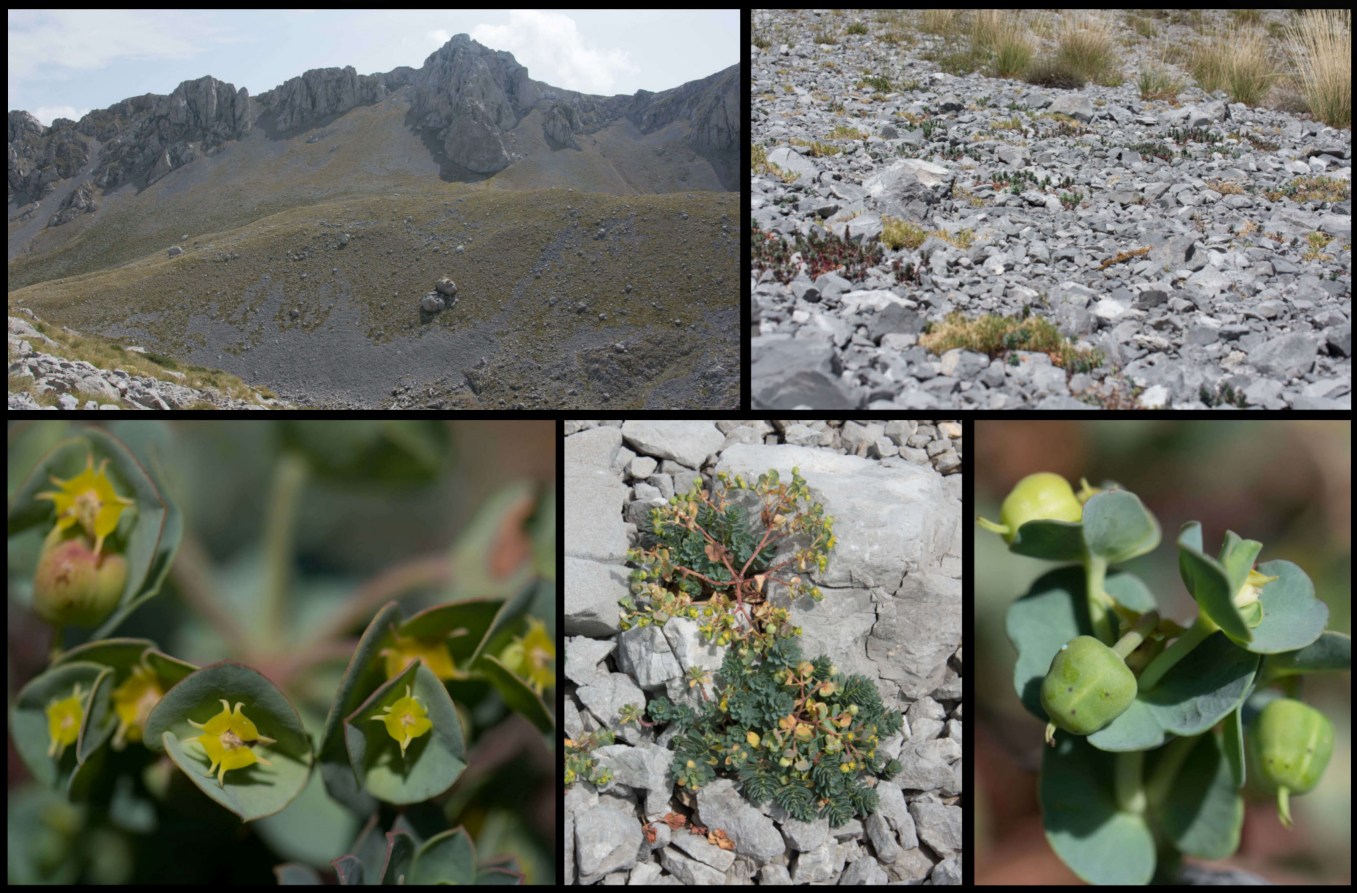
*Euphorbia orphanidis* in its natural habitat on Mt. Parnassos with parts of the inflorescences showing cyathial glands and fruits.

*Euphorbia orphanidis* is distributed in partly mobile to stabilized, mostly south-easterly (one north-easterly) exposed calcareous scree slopes on Mt. Parnassos (**Figures 2**, **7**). Despite visiting and inspecting several scree slopes scattered around the Parnassos Mountain Range, we found the species only in five localized patches south-southeast of the main summit Liakoura (2,457 m). All localities are situated on the slopes of two main valleys separated by the ridge of Mavra Litharia (2,327 m) and Koukos (2,235 m), *i*.*e*., the valley of Velitsa stream southwest of Tithorea and the valley west of Davlia above the monastery Moni Ierousalim. The localities are positioned between 1,500 and 2,300 m, *i*.*e*., above the timberline, which is, in this part of the mountain range, formed by *Abies cephalonica*. Two patches in the former valley are in the area of Chouni south of the main summit Liakoura and one in the side valley above Tsares (slopes of Psilo Kotroni). In the latter valley, one patch is situated just above the timberline in the area of Kanalia, whereas the other is just below Akrino Nero. In all localities, we noted the presence of *E. orphanidis* only in patches of stabilized but open screes with medium-sized screes—in areas with bigger- or smaller-sized screes, the species does not occur.

We could not find *E. orphanidis* in the area around the Ski Club Athens reported by Garnweidner (1986), and no other collectors collected or reported it from this part of Mt. Parnassos. In this area, we only found the morphologically similar *E. deflexa* and noted the absence of suitable habitats for *E. orphanidis*; therefore, we deem this record erroneous. E. Garnweidner (written communication to B. Frajman on 22.9.2020) confirmed that the report of *E. orphanidis* for the summit region of Lyakoura (Garnweidner, 1986) actually was erroneous and corresponded to *E. deflexa*; therefore, both other reports from the very same day published by Garnweidner (1986), *i*.*e*., the one from a karstic depression in the area of the Ski Club Athens and that from the area of the newer ski resort on the northern flanks of Mt. Parnassos are likely erroneous as well. Along the same line, we were not able to locate the historical locality “Lugari”, from which the species was described, as this toponym is not mentioned in the maps and also not familiar to the national park authorities. “Likeri”, a glacial cirque just northeast below the main summit, sounds similar to Lugari and is characterized by extensive screes, but we did not find *E. orphanidis* there.

### 3.4 Ecological characterization of *E. orphanidis* including environmental niche modeling

We registered 31 species accompanying *E. orphanidis* (**Table 1**). Two of them are endemic to Stereá Elláda, six to Greece, and many to the Balkan Peninsula, whereas some are more widespread. Among them, the most commonly found species in several locations along with *E. orphanidis* were *Digitalis laevigata* subsp. *graeca*, *Drypis spinosa*, *Festuca spectabilis*, *Scrophularia lanciniata*, and *Sideritis raeseri*.

**Table 1:** Accompanying species of *Euphorbia orphanidis* on the calcareous screes on Mt. Parnassos (Greece).

Taxon	Distribution
*Aethionema saxatile* subsp. *creticum* Boiss. & Heldr.	Widespread (East Aegean islands, Greece, Kriti, Turkey)
*Anthemis spruneri* Boiss. & Heldr.	Greece
*Thliphthisa purpurea* subsp. *apiculata* (Sm.) P.Caputo & Del Guacchio.	Widespread (Balkan Peninsula and Turkey)
*Carum heldreichii* Boiss.	Greece
*Centaurea musarum* Boiss. & Orph.	Greece
*Cerastium candidissimum* Correns.	Greece
*Cicer incisum* (Willd.) K.Malý.	Greece, Iran, Kriti, Lebanon-Syria, North Caucasus, Transcaucasus, Turkey
*Dactylis glomerata* subsp. *hispanica* (Roth) Nyman.	Widespread
*Daphne oleoides* Schreb.	Widespread
*Digitalis laevigata* subsp. *graeca* (Ivanina) K.Werner.	Balkan Peninsula (Greece, Bulgaria)
*Drypis spinosa* L.	Balkan Peninsula and Italy
*Euphorbia deflexa* Sm.	Albania, Greece, Kriti
*Euphrasia salisburgensis* Funck ex Hoppe.	Widespread
*Festuca spectabilis* Bertol.	Balkan Peninsula and Italy
*Lactuca viminea* (L.) J.Presl & C.Presl.	Widespread
*Linaria peloponnesiaca* Boiss. & Heldr.	Balkan Peninsula
*Lysimachia serpyllifolia* Schreb.	Greece
*Marrubium velutinum* Sm.	Greece
*Melica ciliata* L.	Widespread
*Morina persica* L.	Widespread
*Nepeta parnassica* Heldr. & Sartori.	Greece and Albania
*Odontites linkii* Heldr. & Sartori ex Boiss.	Cyprus, East Aegean islands, Greece, Kriti
*Pterocephalus pterocephala* (L.) Dörfl.	Greece
*Petrosedum ochroleucum* (Chaix) Niederle.	Widespread
*Ranunculus brevifolius* Ten.	Balkan Peninsula, Italy and Turkey
*Sanguisorba minor* Scop.	Widespread
*Scrophularia heterophylla* subsp. *laciniata* (Waldst. & Kit.) Maire & Petitm.	Balkan Peninsula (Balkan Peninsula)
*Sempervivum marmoreum* Griseb.	Widespread
*Senecio thapsoides* DC.	Balkan Peninsula
*Sideritis raeseri* Boiss. & Heldr.	Balkan Peninsula
*Thamnosciadium junceu*m (Sm.) Hartvig.	Greece
*Valantia aprica* (SM.) Tausch.	Albania, Greece, Kriti
*Verbascum graecum* Heldr. & Sartori.	Balkan Peninsula and Turkey

The distribution data are from POWO (2022) and Papiomytoglou et al. (2021), and the nomenclature follows POWO (2022). The distribution data are given for the species and within the parentheses for the infraspecific taxon listed.

**Figure 8:**
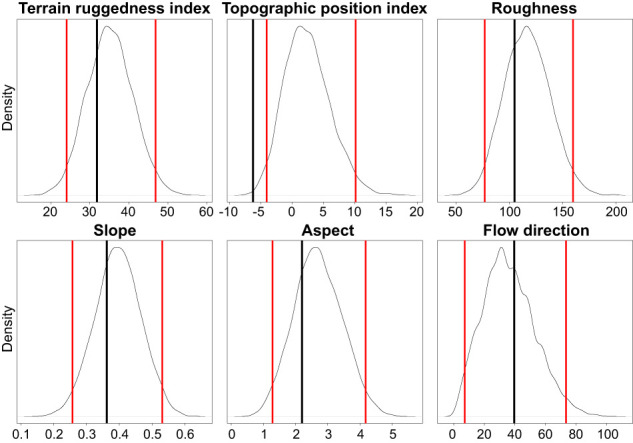
Density distribution of the mean topographic predictors of randomly distributed points (repeated 10,000 times). The red lines depict the 95% confidence interval of these random samples, while the black vertical lines depict the mean predictor values of the populations of *Euphorbia orphanidis*.

From all the topographic predictors analyzed, only the Topographic Position Index (TPI) was significantly different (lower) for the sites were *E. orphanidis* was present in relation to the sites where it was absent (**Figure 8**). This indicates that the localities where *E. orphanidis* was recorded have a concave topography, *i*.*e*., they are at the bottom (small valleys) of scree fields.

## 4 Discussion

### 4.1 *Euphorbia orphanidis* belongs to *E. sect. Patellares* and not *E. sect. Pithyusa*

Our phylogenetic data based on nuclear and plastid sequences clearly show that *E. orphanidis* belongs to *E.* sect. *Patellares* (Prokh.) Frajman and not to *E.* sect. *Pithyusa* as suggested by Geltman (2009) and Riina et al. (2013) based on the species’ morphology. *Euphorbia orphanidis*, along with 17 other species of *E.* sect. *Patellares* (Pahlevani and Frajman, 2023), likely originated in the late Tertiary. Whereas the divergence between *E. amygdaloides* and *E. characias* was dated to the Miocene/Pliocene boundary 5.6 Ma (Horn et al., 2014), our dating analysis rather inferred a younger divergence between these two species as well as among most other species of the section, probably due to a more complete taxon sampling in our study. This divergence was dated to the late Pliocene 2.8 Ma (**Figure 4**), and it is likely that most of the species of *E.* sect. *Patellares*, including *E. orphanidis*, originated in the Pliocene. The aridification that started 9–8 Ma and resulted in the establishment of the Mediterranean climate in the Pliocene 3.2–2.8 Ma (Suc, 1984) triggered the fragmentation of previously forested areas (Milne and Abbott, 2002), which likely caused the speciation in *E.* sect. *Patellares* that predominately includes mesophilous forest species (Riina et al., 2013). Many Tertiary species found a shelter in the Mediterranean Basin that is recognized as one of the most important refugia for species of this ancient flora (Thompson, 2005) and as a biodiversity hotspot with an exceptional role in the preservation of unique species and genetic diversity (Myers et al., 2000; Nieto Feliner, 2014). *Euphorbia orphanidis* certainly holds a special position among these species given its very limited distribution.

*Euphorbia orphanidis* is glabrous and glaucous (**Figure 7**), which fits well to other species of *E.* sect. *Pithyusa*. In addition, ecologically, growing in open, dry, and rocky habitats, it more resembles the members of *E.* sect. *Pithyusa*. In contrast, the majority of species belonging to *E.* sect. *Patellares* are mesophilous and grow in forests and scrublands (Riina et al., 2013; B. Frajman, personal observations). The leaf venation in *E. orphanidis* is not prominent but clearly pinnate, which is characteristic for *E.* sect. *Patellares*, whereas members of *E.* sect. *Pithyusa* have palmate leaf venation. Cyathial glands are semilunate to trapezoid with two horns and thus resemble other species of *E*. sect. *Patellares*, even if some members of *E.* sect*. Pithyusa* also have similar glands. Furthermore, the ovoid trilobed capsules fit better to *E.* sect. *Patellares*, whereas the members of *E. sect. Pithyusa* mostly have conical capsules. However, *E. orphanidis* is the only species of *E.* sect. *Patellares* which does not have connate raylet leaves, a feature that was considered a synapomorphy for this section (Frajman and Schönswetter, 2011; Riina et al., 2013) and likely got lost in *E. orphanidis*. Finally, the RGS of *E. orphanidis* also lies in range of the genome size of other members of *E.* sect. *Patellares* (**Figure 6**), which indicates that it is a diploid species and likely has 20 chromosomes, a typical number for this section (Frajman and Schönswetter, 2011; Riina et al., 2013; Rice et al., 2015); despite several trials, we could not establish the chromosome number for *E. orphanidis*, as no nuclei in appropriate division phase were found.

Contrary to the clear position of *E. orphanidis* within *E.* sect. *Patellares*, its precise origin within this section remains unclear. The ITS tree (**Figure 3**) is unresolved, and most of the species, including *E. orphanidis*, form their own clades in a polytomy. The NeighbourNet is star-like, suggesting a more or less simultaneous divergence among the species. In addition, the plastid phylogenetic trees (**Figure 5**) are largely unresolved but suggest a close relationship of *E. orphanidis* with populations of *E. characias* from the eastern Mediterranean. Even if plastid phylogenies in *Euphorbia* subgen. *Esula* mostly do not follow species boundaries, are commonly geography-correlated, and the populations of different species can share the same or similar haplotypes (Hand et al., 2015; Frajman et al., 2016; Frajman and Schönswetter, 2017), a closer relationship of *E. orphanidis* with *E. characias* appears plausible. *Euphorbia characias* is a widespread Mediterranean species that mostly occurs at lower altitudes compared with *E. orphanidis* but can also reach altitudes of 2,000 m (Lafranchis and Sfikas, 2009; B. Frajman, personal observations); we did not observe this species in Mt. Parnassos, but roughly 50 km away in the Corinthian Bay (B. Frajman, pers. observ.). Within *E.* sect. *Patellares*, *E. characias* is the most thermophilous species, often growing in dry stony garrigues. In this respect, it is the species from *E.* sect. *Patellares* that is ecologically most similar to *E. orphanidis*. Another species from this section that partly ecologically resembles *E. orphanidis* is a high-elevation ecotype of *E. heldreichii* Orph., an endemic species of the southern Balkan Peninsula that mostly grows in thermophilous lowland forests but can extend its range to the alpine belt (Caković and Frajman, 2020). We have also registered it in gravelly open grassland at 2,000 m in Mt. Parnassos. *Euphorbia orphanidis* also differs from both species and other members of *E.* sect. *Patellares* habitually, as it is much smaller than the other species. This is a trait that is generally considered typical of narrow endemics (Lavergne et al., 2004), and our data corroborate this hypothesis.

### 4.2 Narrow distribution of *E. orphanidis* is limited by microtopography and is of high conservation concern

Despite considerable efforts to find *E. orphanidis* in scree habitats scattered across the Parnassos Mountain range, we have only found it in five patches positioned between 1,500 and 2,300 m, in two valleys draining towards the east and positioned south-southeast of the main summit Liakoura (2,457 m). Topological heterogeneity is likely the most important factor influencing the distribution of *E. orphanidis* on Mt. Parnassos. As carunculate seeds of *Euphorbia* species are being dispersed by ants (Espadaler and Gómez, 1997) and several *Euphorbia* species, also those from *E.* sect. *Patellares*, have wide distributions, it seems unlikely that dispersal limitation is the reason for the limited distribution of *E. orphanidis*, but rather its specific ecology. TPI, on the other hand, gives a possible explanation. The only topographic predictor that was significantly different (lower) at sites were *E. orphanidis* is present compared with the screes where we could not find it was TPI. This indicates that the species only thrives in the concave small valleys towards the bottom of scree fields, where snow brought by avalanches accumulates. These sites are therefore likely more humid due to longer snow cover and less water runoff compared with the steeper scree areas above. On the other hand, more flat areas in the surroundings mostly have denser vegetation (B. Frajman, personal observations), which also limits the distribution of weak competitors such as *E. orphanidis*.

Quezel (1964) described an association of *Sclerochorton* (*i*.*e*., *Thamnosciadium*) *junceum* and *Euphorbia deflexa* from the screes between 1,600 and 2,100 m on Mt. Parnassos and Mt. Giona, including further character species as *Freyera parnassica* Boiss. et Heldr., *Galium apiculatum* Sm. (= *Asperula purpurea* subsp. *apiculata*), *Nepeta sibthorpii* Benth subsp. *parnassica* (Heldr. & Sart.) (*= Nepeta parnassica*), as well as *Cicer ervoides* Brign. (= *Cicer incisum*). Based on the differential species *E. orphanidis*, *Festuca spectabilis* subsp. *affinis* (Hack.) Hack., and *Chaenorhinum minus* (L.) Lange, he proposed a local sub-association from Mt. Parnassos, especially from the Gourna region, which further corroborates our results indicating that *E. orphanidis* grows in very specific ecological conditions on Mt. Parnassos that do not occur on the neighboring Mt. Giona, thus likely limiting its distribution also in a broader area, not only in Mt. Parnassos.

Small and genetically depauperate populations with narrow ecological niche are expected to undergo significant reductions in the near future (Theodoridis et al., 2018). Narrow endemic species often grow on slopes with high rock cover and open vegetation (Lavergne et al., 2004), similar to the habitats of *E. orphanidis* and many other Greek endemics. A total of 890 angiosperms of the Greek flora grow in screes, and 46.6% of them are endemic to Greece (Panitsa et al., 2021). Eight of 31 recorded accompanying species of *E. orphanidis* are likewise Greek endemic (**Table 1**), and three of them (including *E. orphanidis*) are endemic to Sterea Elladas (Panitsa et al., 2021). Narrow endemics growing in screes are generally less stress tolerant than their widespread relatives (Lavergne et al., 2004) and are considered weak competitors, thus intolerant to human disturbances (Panitsa et al., 2021).

As Mediterranean high-mountain plants are expected to face extreme heatwaves and summer droughts caused by climate change, which will not only influence the survival and fitness but also trigger changes in the reproduction and regeneration of these plants (Giménez-Benavides et al., 2017), and *E. orphanidis* is not well adapted to drought based on our environmental modeling, we can anticipate a continuing decline in the area of occupancy and extent and quality of habitat of this species in the future decades. This, along with its current area of occupancy being less than 10 km^2^ (**Figure 2**) and its occurrence only in one mountain range (Mt. Parnassos) at no more than five locations, suggests that *E. orphanidis* is endangered (EN) following criterion B of the IUCN classification (IUCN, 2012).

### 4.3 Identification key to *Euphorbia* species on Mt. Parnassos

**Figure 9:**
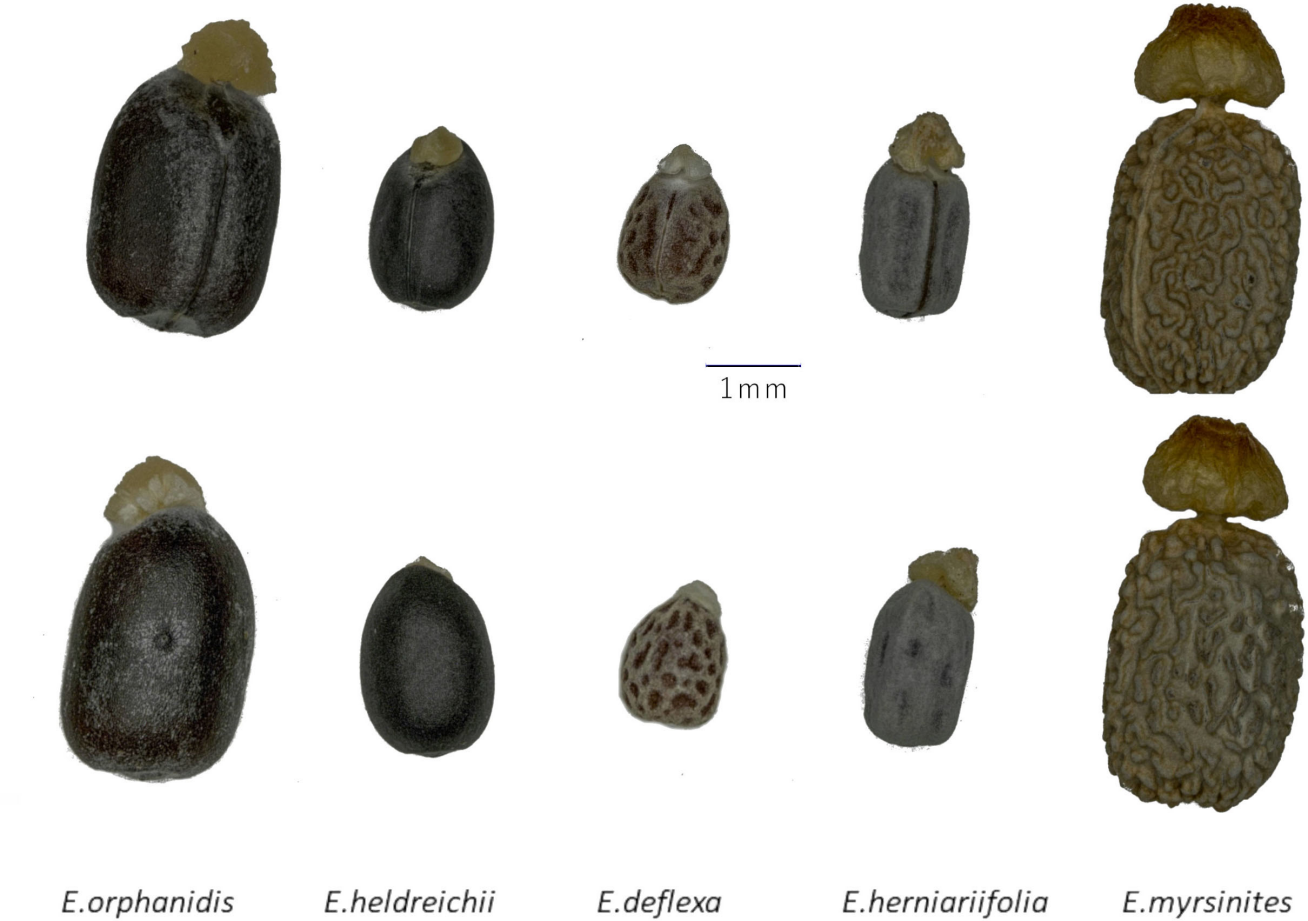
Seeds of five *Euphorbia* species growing on Mt. Parnassus (above, ventral view; below, dorsal view).

*Euphorbia orphanidis* can be confused with other *Euphorbia* species occurring on Mt. Parnassos (see above; cf. Garnweidner, 1986), where they partly grow in similar habitats and co-occur on the same plots, especially *E. deflexa*. To avoid their misidentifications in the future, we provide an identification key for all *Euphorbia* species that we registered above the timberline on Mt. Parnassos, including photographs of their seeds (**Figure 9**).

1 Plant upright, procumbent to ascending, not glaucous-papillose, (30)35–60(80)cm high, leaves (3)4.5–7(10) cm long, raylet leaves connate………………………*E. heldreichii*
1* Plant prostrate, decumbent to ascending, glaucous-papillose, up to 30(4) cm high, leaves up to 2.5(3.5) cm long, raylet leaves free……………………………………………………2
2 Caespitose plant with numerous prostrate to decumbent stems, (2)5–15(25) cm long, usually forming dense mats, without axillary rays. Cauline leaves dense, small, (1)2–5(8) × (1)2–3(5) mm. Terminal rays 2–3. Capsules 2.5–3.5 × 3.5–4.0 mm, with two wings on each keel. Seeds shallowly pitted…………………………………………*E. herniariifolia*
2* Mostly larger plant not forming dense mats, usually with some axillary rays. Leaves bigger, longer than (3)5 mm and broader than (2)3 mm. Terminal rays mostly more than 3. Capsules often larger (but not in *E. deflexa*) not winged on valves. Seeds smooth, vermiculate-rugose or reticulate-pitted……………3
3 More robust plant, with densely leafy stems. Leaves sessile, cuspidate or mucronate, rather thick and fleshy, (10)15–20(35) × (4)7–12(18) mm. Nectarial glands with dilated, often weakly lobed horns. Capsule (4)5–6(7) × 6 mm. Seeds 3.5–4.5 × 2–2.5 mm, vermiculate or rarely smooth……*E. myrsinites*
3* Less robust plant, with less densely leafy stems. Leaves shortly petiolate, not cuspidate or mucronate, not thick and fleshy, (2)5–12(15) × (1.5) 3–6(7) mm. Horns of nectarial glands not dilated, long and slender. Capsule (2.8)3.0–4.5(4.8) × (3)3.5–4.5(5) mm. Seeds 2–2.5 × 1.5–1.8 mm, reticulate-pitted or smooth………4
4 Plant ascending to erect, without fleshy long rhizomes. Leaves (2)5–10(15) × (1.5)3–4(6) mm. Capsule (2.8)3.0–3.2(3.5) × 3 mm. Seeds reticulate-pitted, 2 × 1.5 mm…………*E. deflexa*
4* Plant prostrate to ascending, with fleshy long rhizomes. Leaves 8–13 × (2.5)3–5.7(7) mm. Capsule (3.1)3.4–4.7(4.8) × (3)3.3–4.9(5) mm. Seeds smooth 2.5 × 1.8 mm………*E. orphanidis*

### 4.4 Taxonomic treatment

*Euphorbia orphanidis* Boiss., 1859, Diagn. Pl. Orient. Nov., Ser. 2, 4: 89. ≡ *Tithymalus orphanidis* (Boiss.) Soják, 1972, Čas. Nár. Mus., Odd. Prír. 140: 175. Lectotype (Aldén, 1986): [Greece] “Flora Graeaca Exsiccata No. 407: *Euphorbia hohenackeri* Boiss. et Orphan, nov. sp., in monte Parnassi prope Lugari (rara), Fl. Jun–Jul, alt. 5000–6000’, 4/16 Jul 1854, *Theodorus G. Orphanides* (G00754290-photo)!. Isolectotypes: W 0031038!*, WU 078186!*; photos: G00754364!, HAL0118607!, K000911958!, L0151758!, P00655601!, P00655602! S 13-12930!, WAG0004323!; LE (according to Geltman, 2009).

Other original material (syntypes): Flora Graeca Exsiccata No. 407: Habit in m. Parnassi regione superiore, rara. Alt. 5000´–6000´. 16. Jul. 18??, *Theodorus G. Orphanides* (G00398586!*); Parnass, *Orphan.* (JE00002895-photo)!; De Heldreich Herbarium Graecum normale No. 344: Inter lapides mobiles, in reg. media m. Parnassi (supra Acrino-nero), alt. 4000´-4500´, Aug. 1855, *F. Giucciardi* (G00398585!*, WU 0078185!*; photos: P00655603!, P00655604!.De Heldreich Flora Graeca Exiccata 2967: *Euphorbia hohenackeri* Boiss. et Orphan., In m. Parnassi reg. media, Aug. 1855, *J. Guicciardi* (M0274982!*; photos: C10011249!, G00754289!, K000911957!, L0151757,LD1033460! P00655605)!; Herbarium Willkommii: *Euphorbia hohenackeri* Boiss. et Orphan., In monte Parnasso ad 3–6000’, 16.7.1854, *Orphanides* (W0102305!*); *Euphorbia hohenackeri* Boiss. et Orphan., M. Parnassos, Jul. m. (MO1911900-photo!; neither the collector nor collection year is listed on the label; therefore, it is not certain if this specimen is a part of the original material).

**Figure 10:**
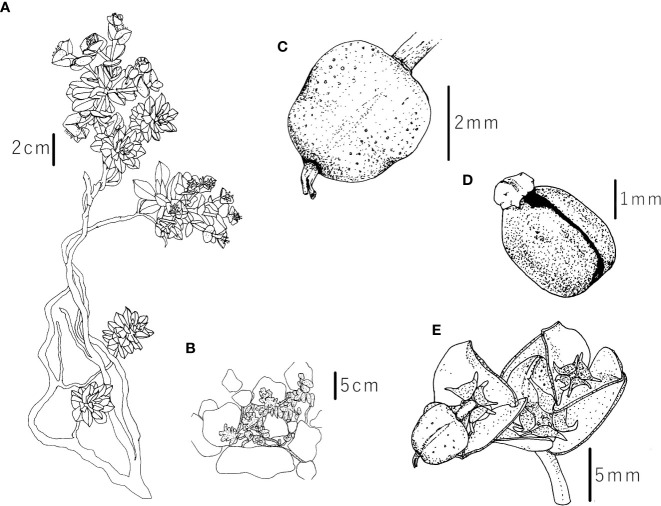
Iconography of *Euphorbia orphanidis*: **(A)** whole plant, **(B)** plant in its habitat, **(C)** capsule, **(D)** seed, and **(E)** inflorescence with fruit and nectarial glands. Drawings by (F) Faltner.

*Description:* Glaucous and glabrous, prostrate to ascending perennial with extensive, branched, fleshy rhizome and several elongated stems. Plant (11)14–26(31) cm high, stems (5)7-20(24) cm long and (1)1.5–3(3) mm thick, with 1–7(11) axillary shoots. Middle stem leaves petiolate, obovate, entire, 8–13 × (2.5)3–5.7(7) mm, (1.3)1.9–3.4(3.7) times longer than wide, widest at (4)5.3–9(11) of the length, with a narrow basis and an obtuse apex. Terminal rays 3–10(11), (7)10–35(80) mm long, 0–6 times dichotomous. Ray-leaves oblong-oblanceolate to ovate-oblong (4)5–8 × (4.5)7.1–12.9(18) mm, (0.6)1.1–1.9(2.2) times wider than long, widest at 0.1–0.4 of their length. Raylet-leaves rhombic-deltate to reniform, obtuse, occasionally emarginate, (2.5)3–6.5(7) × (4.5)5.5–9.5(11) mm, (1)1.2–2(2.2) times wider than long, widest at (0.5)0.7–3(3.5) of their length. Cyathial involucre (1.1)1.2–2(2.1) × (0.9)1–2(2.5) mm, (0.8)0.9–1.1 times longer than wide. Nectarial glands yellow, 1.5–1.6 × 1.4–1.6 mm, with two, 0.7–1 mm long, occasionally bifid horns. Capsule deeply sulcate, smooth, (3.1)3.4–4.7(4.8) × (3)3.3–4.9(5) mm, 0.9-1 times as long as wide, widest at (0.02)0.06–0.3 of the length. Style 0.10–0.13 mm long. Seeds smooth, broadly ovoid and dark grey 2.5 × 1.8 mm, 1.4 times longer than wide, widest at 0.1 of the length. Caruncle (0.6)0.7–0.9 × 0.9–1.3 mm, 1.2–1.6 times wider than long (**Figures 7**, **10**).

*Distribution and habitat*. Endemic to the eastern part of Mt. Parnassos in Central Greece (**Figure 2**). Partly mobile calcareous scree slopes above the timberline from 1,500 to 2,300 m (**Figure 7**).

*Conservation status*. Endangered (EN) according to IUCN (2012) criterion B2.

*Additional specimens seen:* Parnassus – ascent to Gourna, May 1862, *John Stuart Mill.* (photo: K000911959!, P00736822)!; Iter Graecum: In lapidosis regionis abietinae Mt. Parnassi, loco Gurna dicto, rare, 15. Jul 1888, *D. Halacsy* (WU 0126727)!; Flora Graeca: Mt. Parnassos, NE-ENE of Arachova, Scree and gravel on a slope facing SE, 1800-1950 m, 06.07.1975, *Lars Åke Gustavsson 6717* (ATHU 41726 photo)!; Flora Graeca: Sterea Ellas, Nom. Viotias, Ep. Levadhiasi, Mt. Parnassos, SE part c.7 km ENE of Arachova, screes, c. 1700 m, 25.08.1982, *P. Hartvig, R. Franzen & K. I. Christensen 10420*, (G00398584!*); Flora Hellenica: Nom. Viotias, Ep. Levadias, Mt. Parnassos, SE part c.7 km ENE of Arachova, shaded rocks – in scree, 1750–1950 m. Lat: 38°31’N Long: 22°49’ E, 25.08.1982, *Hartvig* et al. *10420* (ATHU 59759 photo!)

## Data availability statement

The datasets presented in this study can be found in online repositories. The names of the repository/repositories and accession number(s) can be found in the article/**Supplementary Material**.

## Author contributions

BF conceived and designed the study, performed field work, coordinated the lab work, performed data analyses, and wrote substantial parts of the manuscript. FF performed field work, parts of the lab work, morphometric measurements, identification of species, and data analyses and wrote substantial parts of the manuscript and used its earlier version as his master thesis. JW performed environmental modeling, wrote corresponding parts of the manuscript, and commented on other parts of the manuscript. All authors contributed to the article and approved the submitted version.
