# Supplementary material for: Phylogenetic data reveal a surprising origin of Euphorbia orphanidis (Euphorbiaceae) and environmental modeling suggests that microtopology limits its distribution to small patches in Mt. Parnassus (Greece)
Source: Front Plant Sci. 2023 Feb 16;14:1116496. doi: 10.3389/fpls.2023.1116496 (PMC9978759; doi:10.3389/fpls.2023.1116496)
Supplement: Supplementary file 1 [file DataSheet_1.pdf]

Supplementary Material

Phylogenetic data reveal a surprising origin of *Euphorbia orphanidis* (Euphorbiaceae) and environmental modelling suggests that microtopology limits its distribution to small patches in Mt. Parnassus (Greece)

Felix Faltner[1], Johannes Wessely[2*], Božo Frajman[1*]

[1]Department of Botany, University of Innsbruck, Sternwartestrasse 15, 6020 Innsbruck, Austria

[2]Department of Botany and Biodiversity Research, University of Vienna, Rennweg 14, 1030 Vienna, Austria

*** Correspondence:**
Corresponding Authors
bozo.frajman@uibk.ac.at; johannes.wessely@univie.ac.at

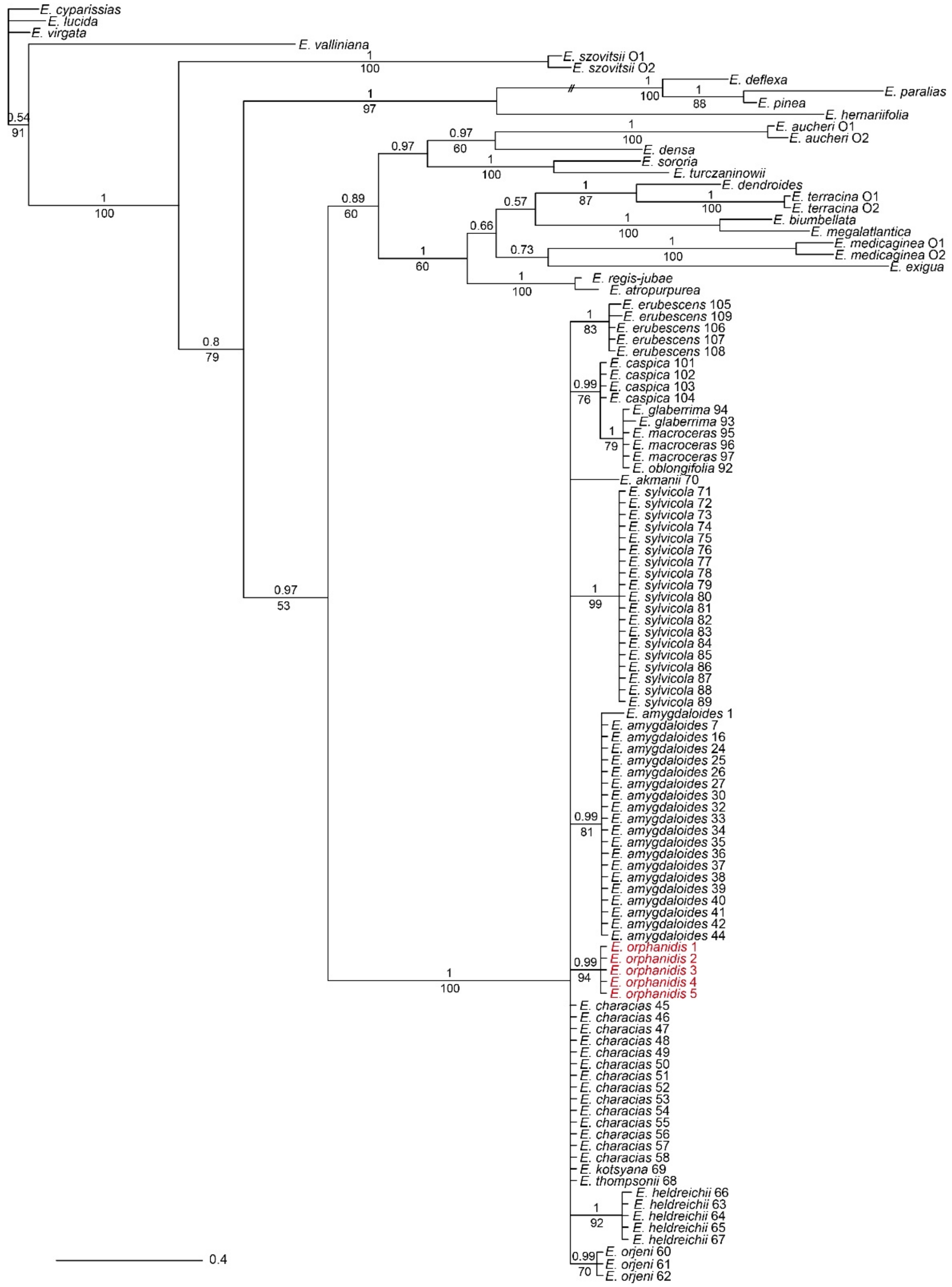

Supplementary Figure 1. Bayesian consensus phylograms of nuclear ITS sequences showing phylogenetic relationships among the members of *Euphorbia* sect. *Patellares* and outgroup species. Numbers above branches are posterior probabilities > 0.5, those below branches are maximum parsimony bootstrap values > 50%. Population numbers of *E.* sect. *Patellares* correspond to Supplementary Table 1 and Fig. 1, those of the outgroup sections to Supplementary Table 2. A simplified version of the tree is shown in Fig. 2.

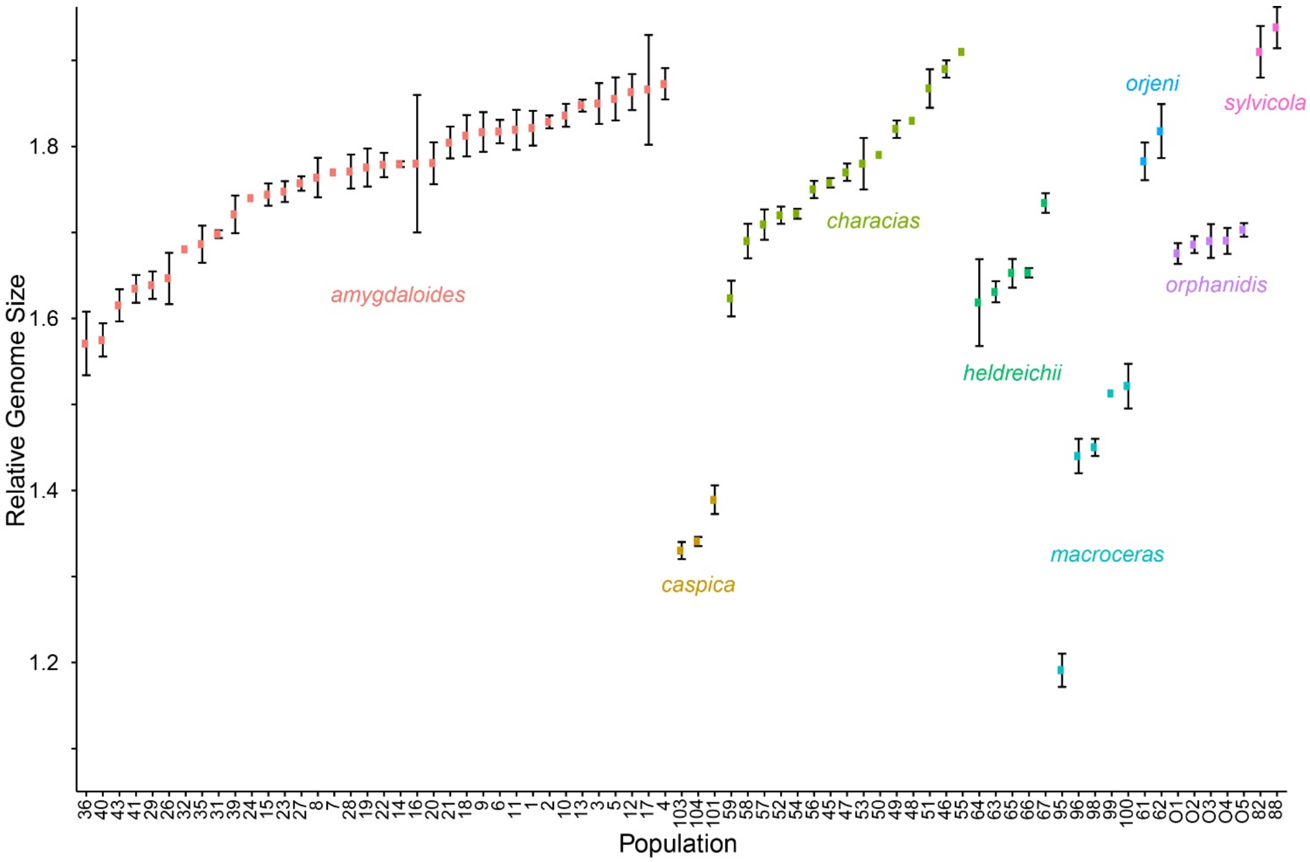

Supplementary Figure 2. Relative genome size (RGS) variation in *Euphorbia orphanidis* and related species from *E.* sect. *Patellares*. Shown are population mean values (dots) and standard deviation (whiskers). Population numbers correspond to Fig. 1 and Supplementary Table 1, where it is also listed how many individuals per population were analyzed. Boxplots of the corresponding RGS values for each species are shown in Fig. 4.
